# Barriers and opportunities to restricting marketing of unhealthy foods and beverages to children in Nepal: a policy analysis

**DOI:** 10.1186/s12889-021-11257-y

**Published:** 2021-07-08

**Authors:** Laura Fisher, Minakshi Dahal, Sarah Hawkes, Mahesh Puri, Kent Buse

**Affiliations:** 1 Indo-Pacific Centre for Health Security, Canberra, Australia; 2 Center for Research on Environment, Health and Population Activities (CREHPA), Kathmandu, Nepal; 3 Institute for Global Health, Faculty of Population Health Sciences, University College London, London, England; 4 Global Healthier Societies Program, The George Institute, Newtown, Australia

**Keywords:** Marketing, Unhealthy foods, Child obesity, Nepal, Policy analysis, Commercial determinants of health, Health policy, Implementation science

## Abstract

**Background:**

Marketing of foods and non-alcoholic beverages high in saturated fats, trans-fatty acids, free sugars, or salt (“unhealthy foods”) to children is contributing to increasing child obesity. However, many countries have not implemented WHO recommendations to restrict marketing of unhealthy foods to children. We sought to understand the absence of marketing restrictions and identify potential strategic actions to develop and implement such restrictions in Nepal.

**Methods:**

Eighteen semi-structured interviews were conducted. Thematic analysis was based on Baker et al.’s 18 factor-framework for understanding what drives political commitment to nutrition, organised by five categories: Actors; Institutions; Political and societal contexts; Knowledge, evidence and framing; Capacities and resources.

**Results:**

All factors in Baker et al.’s framework were reported to be acting largely as barriers to Nepal developing and implementing marketing restrictions. Six factors were identified by the highest number of respondents: the threat of private sector interference in policy-making; lack of international actor support; absence of well-designed and enacted policies and legislation; lack of political commitment to regulate; insufficient mobilisation of existing evidence to spur action and lack of national evidence to guide regulatory design; and weak implementation capacity. Opportunities for progress were identified as Nepal’s ability to combat private sector interference - as previously demonstrated in tobacco control.

**Conclusions:**

This is the first study conducted in Nepal examining the lack of restrictions on marketing unhealthy foods to children. Our findings reflect the manifestation of power in the policy process. The absence of civil society and a multi-stakeholder coalition demanding change on marketing of unhealthy food to children, the threat of private sector interference in introducing marketing restrictions, the promotion of norms and narratives around modernity, consumption and the primary role of the individual in regulating diet - all have helped create a policy vacuum on marketing restrictions. We propose that stakeholders focus on five strategic actions, including: developing a multi-stakeholder coalition to put and keep marketing restrictions on the health agenda; framing the need for marketing restrictions as critical to protect child rights and government regulation as the solution; and increasing support, particularly through developing more robust global policy guidance.

**Supplementary Information:**

The online version contains supplementary material available at 10.1186/s12889-021-11257-y.

## Background

A child watching children’s programming on television in Nepal for an hour a day, every day, for a year would be expected to watch over 30 h of commercials marketing foods and non-alcoholic beverages high in saturated fats, trans-fatty acids, free sugars, or salt (hereafter, “unhealthy foods”^1^) [1]. Television advertising is one of many forms of marketing to which children, defined as those under 18 years [2], are exposed to. Such exposure is problematic as food marketing to children – dominated by marketing of unhealthy foods – has “*a direct effect on children’s nutrition knowledge, preferences, purchase behaviour, consumption patterns and diet-related health”* ([3], p210) and weight outcomes [4] – and the effect is modifiable [3].

Children are particularly vulnerable to marketing as they are not developmentally equipped to assess marketers’ claims or understand marketing’s purpose [5] (such understanding does not fully develop until late adolescence or adulthood [6]). It has been postulated that children in low- and middle-income countries (LMICs) may be more susceptible to marketing than those in high-income countries due to the novelty of marketing in these countries [7].

Notably, the World Health Organization (WHO) Commission on Ending Childhood Obesity has called the evidence linking unhealthy food marketing to childhood obesity “*unequivocal*” ([8], p13) – and the consequences for overweight and obese children are high. They range from adverse health outcomes over the life-course [9, 10] to diminished quality of life, educational attainment and labour market outcomes [8].

Effective policy action to prevent this threat to children’s health is increasingly urgent in Nepal as its health system will struggle to cope without preventative action [11] particularly as: NCDs are estimated to cause 49% of all premature death [12] and most disability [13]; 2.1% of children under five are overweight [14]; and 8.5% of 5–9 year-olds are overweight and 2.6% are obese, while 7.0% of 10–19 year-olds are overweight and 1.7% are obese – figures that have more than doubled in the 10 years to 2016 [15].

While a comprehensive picture of unhealthy food marketing to children in Nepal is not available, we have some understanding of the current terrain. The marketing environment in Nepal is diversified, covering online, outdoor and social network mediums [16]. In a review of multinational companies operating in Nepal, it was reported that some used free toys, cartoon characters and famous people to promote unhealthy foods to children [17]; and in 2016 it was reported that 85% of mothers in the Kathmandu Valley with children under 24 months of age had seen promotions for commercial snack foods [18]. Nationally representative data also show that Nepali households’ food consumption is changing rapidly. Households reported consuming 84% more sugar in 2010–11 compared to 2003–04, while during the same period, there has been an almost tenfold increase in households’ sweets consumption from 16 to 137 g per month [19]. Over a third of 13–17-year-olds also report drinking carbonated soft drinks one or more times per day [20].

In 2010, 192 WHO Member States – including Nepal – endorsed [21] the WHO’s evidence-informed recommendations to reduce *“the exposure of children to, and power of,* [unhealthy food] *marketing”* (WHO Marketing Recommendations) ([22], p8). The recommendations place the onus on governments to determine the most effective approach (e.g. government-led regulation and/or industry-led standards) to reduce marketing to children to achieve the policy objective and to be the key stakeholders in policy development, but ultimately provide flexibility as to the form of regulation.

Despite WHO’s Marketing Recommendations being a WHO non-communicable diseases (NCD) prevention overarching/enabling action for the ‘best buys’^2^ [23] and being endorsed and reaffirmed in United Nations (UN) General Assembly Political Declarations [24, 25], the WHO has described implementation as a “*concerning failure*” ([8], p19). In 2016, Kraak et al., also found that “*No Member State ha [d] implemented comprehensive legislation or enforced mandatory regulations to prohibit the marketing of [unhealthy food] to young people*” [26].

The lack of action in Nepal to develop and implement marketing restrictions is of growing concern [27]. To strengthen Nepal’s response to its growing NCD burden and overweight and obesity among children, it is necessary to understand why Nepal has not developed or implemented policy in line with the WHO Marketing Recommendations – and identify the barriers and potential opportunities to remedy the gap. A review of peer-reviewed and grey literature, and Nepal-specific policy documents, conducted in June 2018 revealed no literature specific to Nepal developing and implementing WHO Marketing Recommendations (see details at Additional file 1).

Our study was designed to fill the evidence gap in Nepal with our overall aim to identify the barriers and opportunities for adoption and implementation of WHO Marketing Recommendations. Drawing on both the findings of our study through stakeholder interviews and a review of the existing evidence base (see Additional file 1) – as well as an examination of power using Lukes’ three faces of power [28] as an explanatory variable influencing factors identified in this study – our second aim was to propose evidence-informed strategic actions that policy-makers and advocates should jointly focus on to promote the adoption and implementation of WHO Marketing Recommendations in Nepal.

## Methods

We undertook a policy analysis including both stakeholder interviews and policy document reviews to understand the current policy response and identify barriers and opportunities in Nepal to developing and implementing a policy to restrict unhealthy food marketing to children. This study design, common in translational health policy research [29], aimed for an in-depth understanding of the drivers of policy-making to combat NCDs, and the potential prospects for future development and implementation of policy to restrict marketing to children [30, 31].

This study which focused on WHO Marketing Recommendations, was a sub-study of a broader study [32] assessing WHO diet-related NCD ‘best buys’ interventions [15] in six countries, including Nepal. Across all six countries we focused on the wide range of policy options for reducing diet-related NCDs, but only undertook in-depth analysis of marketing restrictions of unhealthy foods to children in one country – Nepal. The UCL Research Ethics Committee (11,787/001) and Nepal Health Research Council (NHRC-360/2017) granted research ethics approval for the broader study.

### Policy document review

We undertook a review of policy documents (including legislation) from the health and non-health sectors (e.g. education, media, food and trade) in Nepal, looking for evidence of Nepal planning to develop or implement a policy to adopt and implement WHO Marketing Recommendations. These policy documents included health sector polices such as the Nepal Health Sector Strategy (2015–2020), National Health Policy 2014, National Nutrition Policy and Strategy 2004, Multisectoral Action Plan for the Prevention and Control of NCDs 2014–2020 (MAP), and non-health sector policies like Multi-sector Nutrition Plan II (2018–2022), Food Act 2023 (1967), Trade Policy 2015, Industrial Policy 2010, and Food Regulations 1970 (see Additional file 2 for a full list).

### Stakeholder interviews

Mapping to identify stakeholders with interests in and power to influence development and implementation of WHO Marketing Recommendations and the ‘best buys’ interventions was undertaken with a National Technical Advisory Committee in Nepal, formed for the broader study. Mapping identified a range of individuals in the areas of government, the private sector (e.g. food and beverage industries), media industry, civil society, research, and the medical profession. Purposive sampling from this wider mapping exercise was used to select stakeholders with a focus on marketing, regulatory expertise and/or children’s nutrition [33].

Selected stakeholders were recruited via email and phone, and the 18 who consented to interview were interviewed face-to-face in the Kathmandu Valley in Nepal in August 2018.

A semi-structured interview guide (see Additional file 3) was used with questions based on Baker et al.’s framework for driving political commitment to nutrition which highlights 18 factors organised by five categories [34]:

i. Actors: Nutrition actor network effectiveness, Strength of leadership, Civil society mobilisation, Supportive international actors, and Private sector interference;
ii. Institutions: Strength of institutions, Effective vertical coordination, and Legislative, regulatory and policy frameworks;
iii. Political and societal contexts: Supportive political administrations, Societal conditions and focusing events, and Ideology and institutional norms;
iv. Knowledge, evidence and framing: Credible indicators and data systems, Evidence, Internal frame alignment, and External frame resonance; and Capacities and resources: Strategic capacities, Organisational capacities, and Financial resources.

The 18 interviewees represented the following stakeholder groups: civil service (5 - GOV); international organisations (3 - IO); health research (3 - RES); private sector (2 - PS); media industry (2 - MED); civil society organisations (2 - CSO); and medical profession (1 - DR) (see Additional file 2). Five stakeholders did not agree to participate, as they did not respond or were unavailable, including two from the private sector. Fifteen interviews were conducted in English and three in Nepali using the interview guide and probing where needed - e.g., seeking further detail or explanation of a response, exploring reasons behind a response, or seeking clarity and checking for inconsistencies. All but one interviewee addressed restricting marketing of unhealthy foods to children, and a number of interviews also addressed other ‘best buys’ policies as relevant to an interviewee’s interest and expertise. However, these additional issues are not the focus of this particular study. Six interviews were conducted by SH, MD and LF, and 12 were conducted by MD and LF. Interview questions were tailored to focus on the area/s of a stakeholder’s interest, expertise, or responsibilities. Many respondents drew from their experience in other areas of policy development in Nepal – e.g. tobacco control – to identify lessons relevant to the aim of introducing marketing restrictions. These findings are presented in our results section where they provide valuable insights into potential strategies and tactics used to achieve policy aims.

Interviews were digitally recorded with permission and interviewers took detailed notes and conducted preliminary data analysis shortly after all interviews. After transcription and checks for accuracy against recordings, interviews were reviewed by LF in NVivo software to enable easier storage, structuring and analysis of data [35].

Theoretical thematic analysis, using Baker et al.’s framework [34], was used to analyse data from the interviews [36].

## Results

We found no policy documents from the health and non-health sectors (see Additional file 2), which addressed Nepal marketing restrictions except vague commitments in Nepal’s MAP [37] which lists implementing WHO Marketing Recommendations as a ‘priority action’, but milestones only include action on marketing of breast milk substitutes (where Nepal has already banned all advertisements and promotions to the public and health care practitioners [38]). Additional relevant actions, such as legislating to ban foods high in trans/saturated fat including their sale around school premises, have not been implemented [37].

All 18 factors in Baker et al.’s framework [34] were reported by respondents as acting largely as barriers to driving political commitment to develop, adopt and implement WHO Marketing Recommendations in Nepal. The six factors that were mentioned by the highest number of respondents (14 or more) as either barriers or opportunities to policy development and implementation are detailed below. Table 1 summarises barriers and opportunities identified by the respondents across the remaining 12 factors in Baker et al.’s framework (13 or fewer respondents identified these factors).

**Table 1:** Summary of other identified barriers and opportunities to developing and implementing marketing restrictions

Factor	Theme identified (area of respondent)
Nutrition actor network effectiveness	- Absence of actors collectively advocating for change (e.g. leaders, institutions) (2 GOV, RES, 3 IO, DR) vs examples where this has occurred for alcohol restrictions (GOV), MAP development (2 RES), and Multi-sector Nutrition Plan policy community cohesion (IO) - Lack of a forum or network where actors come together to address NCDs (GOV, MED, RES) - People, social leaders need to be vocal and consistently advocate and press for change (RES, CSO) - Based on legalising abortion, need ground up push so Government cannot say no (RES)
Strength of leadership	- No strong leadership on NCDs, and even less on risk factors for NCDs (GOV, IO) - Health Minister has failed to take a lead; if there is a powerful leader, things get done (2 GOV, RES), e.g. during MAP development (RES) under the previous minister (IO)
Civil society mobilisation	- Lack of civil society debating and lobbying on NCDs (IO, GOV); NGOs should provide suggestions, put pressure on government to push for policy formulation (2 MED) - Civil society proved important in resisting private sector interference, such as in tobacco (GOV), and in pushing for stronger action from the Nepal Press Council on media content (MED). One CSO respondent considered NGO advocacy along with that of clinicians talking about the rise in the NCD burden in Nepal critical to the government formulating the MAP. - Nepal NCD Alliance talking about the need for action on NCDs (RES), and various other civil society, such as the Nepal Heart Foundation and Nepal Diabetes Society, but they are active in their own spheres, not active as a coordinated group (CSO)
Strength of institutions	- Lack of multisectoral coordination for the MAP across Ministries (RES, 3 GOV, 2 IO) with for example the high level NCD Committee having only met once since 2014 (IO) - Inadequate government structures (2 RES, 2 IO, GOV, CSO), even though a designated structure for NCDs has been established within the MoHP post federalization (RES) - MoHP has the power for bilateral action, but potentially lacks mandate for multisectoral action (RES, GOV); but some consider that MoHP should lead (GOV, RES) - No one leading on MAP implementation (but everyone is involved), unlike the Multi-sector Nutrition Plan which was led by the National Planning Commission (IO); National Planning Commission should be the executing body for the MAP as many elements outside the control of health, similar to its role in nutrition (2 GOV, IO)
Effective vertical coordination	- Lack of vertical coordination for the MAP between policy responsibility at the MoHP and implementation by the Department of Health identified, noting that this was not the case previously, potentially due to changed leadership (RES) - Need for regional cooperation to deal with cross-border issues (PS) - Previously demonstrated successful vertical coordination within and external to government in iodisation efforts to address goiter (PS) and in food standard setting (GOV); but also inadequate vertical coordination in implementing tobacco control laws where for example tobacco officers regulating the law do not understand their roles and responsibilities (GOV)
Societal conditions and focusing events	- Attention diversion at time of the MAP (e.g. the earthquake and federation reform and lack of stability) could have impeded development and implementation (2 GOV, CSO) - Crowded government agenda in health, including the unfinished Millennium Development Goals and the Sustainable Development Goals (IO) - Cross-border media and imports of food from India and China (CSO, PS, IO, DR) create issues in regulating (MED), including due to Nepal’s World Trade Organization membership (MED) - Junk food advertisements from India, e.g. influence of Delhi life, is contributing to changing social attitudes among school students who ridicule home-made foods (GOV) - Now is the time to act (MED), as overweight and obesity has more than doubled in the last 15 years, and access to and use of unhealthy foods has increased (IO, GOV, RES)
Ideology and institutional norms	- Prevailing beliefs of the need to have sugar in diet to survive (CSO) or that children should eat noodles to ensure they are not malnourished (more common in rural areas) (GOV) - May encounter public resistance in trying to restrict items like biscuits and noodles that have meaning for common people / where strong beliefs around nutrition (PS, DR)
Credible indicators and data systems	- 5-yearly NCD Risk Factors STEPS survey established to provide clear data and indicators on NCDs (IO, GOV, RES) - Food consumption surveys have not been undertaken to distil eating patterns in Nepal – should be done, but there is insufficient capacity (GOV); and only scattered efforts on indicators specifically on the degree of the problem of marketing unhealthy foods to children (DR)
Internal frame alignment	- Some recognition among those involved in the policy process of the importance of NCD prevention (and regulation) rather than just a treatment, but they consider (mistakenly) that the most effective approach is behaviour change (RES, CSO, IO, GOV) - Others considered that structural policies, including marketing restrictions, are the right approach to achieve the most progress, alongside behaviour change (RES, CSO)
External frame resonance	- Framing marketing restrictions as an issue of child rights could have the power to convert to action / draw the government’s attention (2 RES, 2 MED, CSO) - Public consider taste and quality of high salt noodles to be more important than health concerns, a view shared by Nepalese industry (GOV)
Strategic capacities	- Evidence of engagement of stakeholders in policy-making, such as through consultation in MAP development (GOV), and facilitating solutions via engaging communities and industry by other key governmental departments or regulators such as the Department of Food Technology and Quality Control via subcommittees (2 GOV) - Lack of strategic capacity indicated: when the MAP was being developed given no one briefed the Chief Secretary and the Prime Minister’s Office on what was expected from them to enable implementation (RES); the government needing to convince the relevant stakeholders – media, advertisement association, and Nepal Press Council – that advertisements are not just for money generation, but also for awareness creation (MED)
Financial resources	- Insufficient funds (DR, CSO), with money from the tobacco tax not being spent on NCD prevention, but only on treatment (CSO) - Government’s expenditure on NCDs in the health budget is too low (< 6%) (RES), and it should go up; should spend funds from alcohol, tobacco, and other taxes on NCDs (IO) - Ministry of Finance needs to be engaged to ensure sufficient resources (IO, RES) - May be fiscal space for policy development in the future (IO)

Acronyms for respondents: civil service (GOV); international organisations (IO); health research (RES); private sector (PS); media industry (MED); civil society (CSO); and the medical profession (DR)

### Private sector interference

Most (*n* = 14) respondents from all sectors spoke of private sector interference in tobacco, alcohol and salt control, and/or considered it would occur if the government introduced restrictions on marketing of unhealthy food to children, or broadly stated that industry did not wish to lose profit.

*“If we initiate making policy on junk food then manufacturers will try to influence it. They will first try influencing at political level. This has happened in case of tobacco…but they could not succeed as our political commitment on tobacco is high”.* (Interview 8, GOV)

One respondent described the role of private sector influence in regulating unhealthy foods:

*“government came up with a policy that unhealthy snacks … should not be allowed in the school premises. Two or three weeks later all those big industries people went to the government and said it is not that bad and also influenced government by explaining about their social contributions. The policy was not taken back officially but was not implemented either”.* (Interview 16, RES)

One private sector respondent stated that self-regulation is the only path available as,

*“If some mandatory regulations are imposed all of a sudden, it won’t work. It must be self-regulatory”.*

(Interview 5, PS)

However, one respondent from a private sector media organisation stated that regulation is needed and that they believed the advertising association would support regulation of marketing to children, not oppose it:

*“Media is a medium to provide information and … guide society. Advertisements are not only for making profits, these can also be medium for raising awareness. Monitoring of advertisements is a must and advertisement board is very much necessary in the country [to] … regulate the kind of advertisements broadcasted to children as well as other population”.* (Interview 18, MED)

### International actor support

Most (*n* = 10) respondents across nearly all sectors noted the lack of broad international support for action on NCDs aside from tobacco control. Three specifically noted the need for a greater global “*mandate*” or “*push*” to generate political commitment for action on diet-related risk factors, including WHO Marketing Recommendations, similar to that for tobacco:

*“As we have already signed [Framework Convention on Tobacco Control] so to control tobacco is one of the top priorities. But there is no such type of treaty in the junk food/processed food, so government is not compelled to focus on these areas”.* (Interview 8, GOV)

Further, seven respondents mainly cited WHO as the *only* international organisation engaged in NCDs; and lack of, or ‘negligible’, international funds and focus; one mentioned lack of regional cooperation. Critically, three international organisation respondents stated that WHO Marketing Recommendations are not a priority among international organisations in Nepal, while one CSO respondent observed that:

“*there are partners, international organisation interested to invest in the control and prevention of NCDs but until and unless the government puts this in the priority agenda nobody is going to come up*”.(Interview 17, CSO)

### Legislative, regulatory and policy frameworks

Five respondents noted that Nepal’s MAP implementation has been weak compared to the Multi-sector Nutrition Plan which involved:

“*serious planning … and a good sum of budget … allocated for its operation*”.(Interview 9, GOV)

MAP implementation has also largely focused on NCD treatment, and tobacco and alcohol control, with three respondents stating that there have been no (or few) “policies” developed on unhealthy food consumption, rather the focus is on behaviour change:

*“There has been some mass media campaigns on diet and reduction of consumption of salt, sugar and transfat. Nothing else … has been done”.* (Interview 1, GOV)

More specifically, respondents outlined three factors that would make developing and implementing WHO Marketing Recommendations in Nepal difficult: 1) Nepal’s weak advertising monitoring and censorship on unethical marketing and misinformation; 2) difficulty in developing and implementing national marketing restrictions given cross-border media, imports and trade rules as Nepal is a member of the World Trade Organization; and 3) given complexity in determining which unhealthy foods would be captured by restrictions. However, others noted: that the Ministry of Communication and Information Technology might be establishing an advertising [regulatory] board; that some schools are implementing bans on unhealthy food sales; and the effectiveness of banning alcohol and tobacco advertisements in electronic media. Further, others advocated the need for regulation of unhealthy food marketing to children to include penalties (citing the example of the effectiveness of penalties for drink driving); and five respondents from the media industry, research and civil society thought regulation could or should be framed as a child’s rights issue.

### Political administration support

A minority of respondents (*n* = 4) noted government support for the MAP development and increasing political commitment on NCDs. In contrast, most (*n* = 14) noted a lack of political support and/or leadership from government for *regulating* NCD risk factors, including regulating the marketing of unhealthy food marketing to children. A range of reasons were suggested that might account for this lack of attention to the regulation of NCD risk factors. First, frequent changes in ministers resulting in lost momentum (two respondents). Second, lack of political support across the spectrum, such as from the Ministry of Finance – contrasted with the Multi-sector Nutrition Plan led by the National Planning Commission, which was a “*major political and health agenda for Nepal*” (Interview 7, IO; four respondents). Third, relative priority of other issues:

“*[the] state may feel the top-most priority in present situation is federal restructuring*”.(Interview 18, MED)

Fourth, a concern with short-term political returns from treatment rather than dispersed long-term rewards for prevention activities (five respondents):

“*if we invest in the prevention now means we can see the outcome in the future after a long time, but if they invest in … immediate treatment … they get the popularity*”.

(Interview 4, GOV)

Nonetheless, the current government’s power to act was noted and presents what is thought to be an important opportunity:

*“this government [has a] … majority and this is one of the strongest … government [s] … if the government wants … they can really enforce … regulation”.* (Interview 12, IO)

### Evidence

Nearly half (*n* = 8) of respondents (largely non-governmental) reported a lack of awareness in the population (particularly amongst less educated or those in rural areas) and among “bureaucrats” in relation to unhealthy foods (in comparison to tobacco), and/or noted poor awareness of structural interventions including WHO Marketing Recommendations. However, nearly half (n = 8) of respondents representing nearly all sectors considered that the government and/or policymakers and general public, recognised the evidence of the NCD problem – but that knowledge had not spurred action:

*“it’s not that government does not know. But there are still communicable diseases … to be tackled”.*(Interview 10, IO)

*“I feel Nepal Health Research Council was not able to convince/drive policy makers … for taking concrete actions based on the … [NCD Risk Factors] STEPS survey”.*

(Interview 9, GOV)

A number of respondents called for further evidence, including: better evidence to justify regulation to industry and analysis on solutions; better return on investment evaluations, such as those that motivated action in undernutrition; and calls, largely by researchers, for greater local evidence, with one stating that political leaders:

*“really don’t want to see any evidence from outside … They always ask for local evidences … whether even the local evidence are used properly”.*

(Interview 11, RES)

However, five respondents agreed that the NCDs ‘best buys’ interventions, including WHO Marketing Recommendations, are evidence-informed and effective:

*“These things have … been proved by experience of other countries where it has been implemented. Restricting these things in the community through policy is effective”.*

(Interview 17, CSO)

### Organisational capacities

Most (*n* = 13) respondents across all sectors captured the concept of weak policy implementation capacity but strong policy-making capacity, even though this is yet to be mobilised to implement WHO Marketing Recommendations. This appeared to be the result of a few factors which were consistently reported, particularly: inappropriate organisational arrangements to lead implementation of and govern marketing restrictions; inadequate human resources, especially those with experience due to frequent restructuring and transfers across “vital posts” and overcrowded agendas; and inadequate monitoring capacity, especially reported in relation to marketing restrictions, where one respondent noted that the Government:

*“don’t have capacity to regulate [international and online media]”.*

(Interview 18, MED)

However, one researcher noted that the Government:

*“should choose the right person...The government brings people from outside who are not aware of the local realities. The … consultant comes and does his job and the thing doesn’t move forward … as … there is no local ownership. Be it abortion, be it women’s health, be it smoking, everything was done by us” (where respondent was reporting on successfully implemented health interventions).*

(Interview 16, RES)

Further, another researcher noted that an opportunity may arise to develop marketing restrictions as more technical people move into the Ministry of Health and Population (MoHP) through federal restructures:

*“they’re trying to get benefit of the technical people being involved in the policy and guidelines level thing, so maybe things can be different now”.*

(Interview 11, RES)

## Discussion

We found an absence of WHO-recommended restrictions on marketing unhealthy foods to children in Nepal. Our review of policy documents found only broad language recommending that marketing restrictions should be in place, but no clear policy response and no identified timeline to develop, adopt and implement the WHO-recommendations.

Our interviews with key stakeholders identified a range of factors that will potentially either limit or enable the development, adoption and implementation of WHO Marketing Recommendations on unhealthy foods to children in Nepal. The factors largely align with the categories set out by Baker et al. [34], and as with Baker’s findings, we have noted that the factors are frequently inter-dependent and change over time. For example, respondents noted if there is political leadership (i.e. to put marketing restrictions on the agenda), support from international organisations will follow. The six most commonly reported factors in this study also align with elements required to achieve healthy food environments [39] and mirror constraints identified by the UN Secretary-General [40] in overall NCD policy-making.

The absence of marketing restrictions and the lack of any clear commitment to develop, adopt and implement the WHO Marketing Recommendations reflects, we believe, the manifestation of power in policy processes. Political theorist, Lukes, proposed that power is exercised in three ways [28]: decision-making power (control over deciding among policy options), non-decision-making power (keeping issues off policy agendas), and ideological power (influencing whether or not and how people think about issues). We believe that all three dimensions in the exercise of power are relevant to understanding our findings.

### Power as decision-making: the role of civil society and multi-stakeholder coalitions in achieving policy change

Alliances and networks between academia, civil society, the medical profession, education sector and media have been highlighted as a key factor in driving political commitment and action, including on children’s nutrition. While individual stakeholders likely exercise low levels of power that political leaders may respond to (or ignore), the combined force of a coalition of actors can be substantial. For example, Baker et al. previously noted that *“[t] he main core action [in political commitment for nutrition] … is sustained commitment-building … by … [Nutrition Actor Networks]”* ([34], p12]. And many respondents noted the previous success of advocacy coalitions in driving policy change in Nepal, including in health (GOV, 2 RES, IO).

The absence of any such coalition in demanding change on marketing unhealthy foods to children may be contributing to Government inaction in this area. A successful coalition would gain ‘power as decision making’ to indirectly influence the policy process and penetrate formal decision making required by the Government of Nepal and act as a countervailing force to the food sector (see Strategic Action 1, below).

### Power as non-decision making: role of private sector interference in inhibiting policy consideration

This real and perceived threat of private sector interference in the introduction of marketing restrictions was noted by one respondent who reported a meeting between ‘big industry’ and government, influencing their views and leading to the reported poor implementation of bans on unhealthy snacks in schools.

The power to keep an issue off the agenda (or under-resource the implementation of the policy) can be as important as the power to see an issue addressed in the policy sphere. Numerous studies in LMICs have found that private sector interference is *“[r] epeatedly … the first and foremost barrier”* to implementing WHO Marketing Recommendations, and that there is substantial uniformity in interference ([41], p5, [42, 43]). Interference has included: lobbying [44–47]; private sector engagement in policy-making [44]; and the establishment of public-private partnerships [47]. Such action has precedent in Nepal. Indeed, nearly 80% of respondents reported private sector interference in tobacco, alcohol, and salt control, and/or that private sector interference *would* occur if the government introduced restrictions on marketing unhealthy foods to children.

### Ideological power: role of norms and narratives in inhibiting policy alternatives

The power to shift the narrative can be exercised in a number of ways. For example, several respondents were sceptical about the quality of empirical evidence on the impact of marketing restrictions. Respondents also indicated that there was a lack of awareness of the health and broader consequences of unhealthy food consumption (including in children) and the efficacy of structural interventions. A diverse range of respondents, including from research, government and international organisations, considered behaviour change to be more effective, despite evidence to the contrary [48]. This shift from structural to individual level responses reflects an ongoing power imbalance in public health narratives, with neoliberal ideologies tending to focus “solutions” at the level of individual behaviour change – despite the lack of empirical evidence for population-level impact [49].

The government’s lack of attention to marketing restrictions could be influenced by reported complexities in such restrictions which arise from cross-border media and food imports from India and China, as well as Nepal’s World Trade Organization membership or trade agreements that Nepal is party to – which may be real and/or perceived complexities. Both of the above explanations for not formulating restrictions could also be influenced by shifts in underlying values. This includes the reported: influence of ‘Delhi life’ which is contributing to changing social attitudes among school students and a rejection of traditional, healthier Nepalese foods (seen in interviews with Nepalese children where a child stated a preference for *“Lays [chips] and coke”* over cooked food [50]); longstanding beliefs in the ‘need’ for sugar and other foods such as noodles in children’s diets for energy and to ensure children are not malnourished; and potential to encounter public resistance in seeking to restrict marketing of unhealthy foods like biscuits and noodles that have meaning for ‘common people’. These responses suggest that norms and narratives promoted around modernity, consumption and the primary responsibility of an individual in regulating diet via behaviour change and education (rather than of government regulation of commercial interests [51]) are playing a role in the policy vacuum on marketing restrictions in Nepal.

### Limitations

The findings of this study may have been biased as only one author (LF) coded interview data [29]. Qualitative interviews were limited in number by time constraints in Nepal and availability of stakeholders. Interview data could be skewed by smaller numbers of some sector-representative respondents, such as those from the private sector. Findings could be subject to respondent recall bias or social desirability bias in considering views on health policies when responding to a health policy researcher. Findings are also limited by the fact that there is only a small community of stakeholders in Nepal with an interest in or power to influence development and implementation of WHO Marketing Recommendations and results may be more representative of barriers and opportunities for broader NCD policy development and implementation. Further, findings are temporal, particularly given often-changing positions reported by respondents within government in Nepal, and often-changing external circumstances affecting policy environments [52].

## Conclusion

This study found that the development and implementation of WHO Marketing Recommendations to restrict marketing of unhealthy foods to children was hampered by a range of factors, illustrative of the distribution and exercise of different levels of power in the policy process in Nepal. These factors included the threat of private sector interference, lack of international assistance, and an absence of political administrative support and multi-stakeholder coalitions pushing for evidence-informed policies and legislation. These findings were compounded by a lack of awareness among our interviewees of both the consequences of marketing of unhealthy foods to children and the effectiveness of restricting marketing.

To move this agenda forward in Nepal we suggest that advocates and policymakers focus on five strategic actions – see Table 2. The most critical of these is forming a multi-stakeholder coalition to generate political incentives to put and keep marketing restrictions on the political agenda. We suggest that such a coalition could have a range of positive mobilising effects, including building multi-sectoral support across ministries critical to developing and implementing marketing restrictions. The international global health community also has a role to play through developing more robust global policy guidance to support development of national regulation, and through providing adequate financial and technical assistance, to “*empower lower- income countries to exercise … sovereign authority to protect the health of their populations*” ([71], p10), including children.

**Table 2:** Strategic action recommendations for promoting evidence-informed policy responses to restrict marketing of unhealthy foods to children in Nepal

Based on the findings from our interviews and wider understanding of the literature on policy change, we have identified five areas where strategic action is needed to generate political incentives to promote the development, adoption and implementation of effective policies to restrict marketing of unhealthy food to children. 1. **Build a multi-stakeholder coalition.** Nutrition advocates, researchers and civil society, should develop a multi-stakeholder coalition to generate political incentives to put and keep marketing restrictions on the political agenda, and counter private sector interference. Nepal has a strong history of Government and civil society collaboration on tobacco (an area in which Nepal has shown policy leadership [53]), and it already has a base of support, such as via the Nepal NCD Alliance and Nepal Heart Foundation. Nepal has also experienced NCD policy success when the policy community (e.g. government, international organisations, research institutions and clinicians) cohesively advocated for evidence informed, NCD policy formation, including in implementing alcohol restrictions and the Multi-sector Nutrition Plan. Civil society mobilisation has also been a key factor in overcoming food industry interference or generating government commitment [41] for marketing restrictions in other countries, via communications networks and media [54], providing technical and financial capacity, or acting as “knowledge brokers” [43, 55]. 2. **Reframe the challenge and use local evidence.** A multi-stakeholder coalition could raise the importance of marketing restrictions by: a. Framing the challenge (unhealthy food marketing) and solution (marketing restrictions) as protecting child rights to justify government intervention to combat recently introduced but increasingly dominant norms and narratives. The child rights frame was suggested by respondents to have the power to draw the government’s attention and get it to convert words into robust regulatory action. A child rights frame has been advocated for globally to help build political will as child rights are often a government priority [56]. As child rights are enshrined in Nepal’s constitution [57] any regulation of marketing of unhealthy food to children in Nepal could rely on these rights [58] and leverage human rights monitoring mechanisms [55]. b. Leveraging existing evidence to show that now is the time to restrict marketing, including evidence of Nepal’s growing NCD burden and of the population’s increased access to unhealthy foods. International evidence of the need for and effectiveness of marketing restrictions, including the most effective form of regulation, is likely to be applicable, and was successfully applied in Chile alongside local evidence [56]. Existing tools could also be used, such as the South-East Asian WHO Region’s Nutrient Profiling Model [59] to provide objective criteria for unhealthy food marketing and ensure proportionate regulations [43, 60]. However, advocates will need to support the generation of new evidence to help design effective marketing restrictions (e.g. to better understand the exposure and power of marketing to children in Nepal). 3. **Adopt a whole of government approach.** The above two actions would help to build political support across the ministries required to develop and implement restrictions on marketing of unhealthy food to children, from the Ministry of Communication and Information Technology to the Ministry of Finance. Brazil’s experience (where the Attorney General suspended a proposal to restrict marketing supported by the ministry of health) suggests whole-of-government support is required [61]. This mirrors experiences in LMICs, where inadequate political administrative support was a barrier to implementation due to governments’ resistance to the hard policy tool of regulating, concern about trade threats, or viewing restrictions as contrary to economic development [41, 47, 55, 62]. 4. **Appoint a lead institution.** An institution with a broad remit should be charged with overseeing policy development and implementation of restrictions via an interagency mechanism. In contrast to the Ministry of Health, the National Planning Commission, with proven experience in multi-sectoral policy execution and high standing among Ministries could more ably deal with the complexity of regulating cross-border marketing, coordinate multi-sectoral action, address competing and norms and narratives, and counter private sector interference. The importance of a strong lead institution was supported by Nepali respondents as well as experience in Thailand where lead agencies needed sufficient authority to operate effectively to implement marketing restrictions [45]. A strong lead institution may also safeguard against any leadership vacuum created by high ministerial turnover, and ensure stronger implementation capacity, including adequate funding and human resources to govern marketing restrictions (reported by respondents and in the literature [63, 64]). 5. **International support.** The above agenda would arguably be aided by: a. A strong international mandate or code for unhealthy food marketing to children (or extension to the International Code of Marketing of Breastmilk Substitutes) - which the Framework Convention on Tobacco Control provided to national tobacco control efforts - has been called for by academics since 2011 [65–67] In 2019, experts also called on the WHO Director-General and the UN High Commissioner for Human Rights to develop human rights guidelines on healthy diets, which included a focus on marketing of unhealthy foods to children [68]. b. In the absence of a global code, greater international financial and technical support for Nepal, including for regional coordination, is needed. It is clear from respondents, and literature from Nepal, the South-East Asian WHO Region, and LMICs that current support is insufficient, including research funding for NCD prevention and control [12, 43, 69, 70]. Such support is a key assumption in Nepal’s MAP [37]. The importance of it has been demonstrated in Mexico which collaborated with the Pan American Health Organization’s task force to control food marketing to children and adolescents [54], and in Fiji, where the WHO provided legal and health expertise to ‘build momentum’ to advance a bill seeking to restrict marketing of unhealthy foods to children [60].	

## Supplementary Information

**Additional file 1.** Literature Review Methodology (Outline of literature review methodology, including search flow chart and document search database).

**Additional file 2.** Respondent Database (List of study respondents and respondent type) and Policy Document Review.

**Additional file 3.** Interview Guide for Stakeholders.

## Data Availability

The datasets used and/or analysed during the current study are available from the corresponding author on reasonable request. The data are not publicly available due to them containing information that could compromise research respondent confidentiality/consent.

## Abbreviations

LMICs: Low- and middle-income countries
MAP: Multisectoral Action Plan for the Prevention and Control of NCDs 2014–2020 [71]
MoHP: Ministry of Health and Population
NCD: Non-communicable diseases
Unhealthy foods: Unhealthy foods and non-alcoholic beverages - high in saturated fats, trans-fatty acids, free sugars, or salt
UN: United Nations
WHO: World Health Organization

## Footnotes

1 We have used the term “unhealthy foods” which is the term frequently used by UNICEF – e.g. in https://www.unicef.org/csr/files/A_Child_Rights-Based_Approach_to_Food_Marketing_Report.pdfCovering the same groups of foods, WHO most frequently uses the term “high fat, sugar and sodium/salt” (HFSS),

2 The ‘best buys’ are the most cost-effective and feasible interventions for policymakers to address the four key risk factors for NCDs (including unhealthy diet), and four disease areas (cardiovascular disease, diabetes, cancer and chronic respiratory disease), along with other recommended interventions to address NCDs. The interventions are to assist WHO Member States in implementing measures to help achieve Sustainable Development Goal Target 3.4 and are part of the Global Action Plan for the Prevention and Control of NCDs 2013–2020.
