# Supplementary material for: Barriers and opportunities to restricting marketing of unhealthy foods and beverages to children in Nepal: a policy analysis
Source: BMC Public Health. 2021 Jul 8;21:1351. doi: 10.1186/s12889-021-11257-y (PMC8268610; doi:10.1186/s12889-021-11257-y)
Supplement: Supplementary file 1 — Additional file 1. Literature Review Methodology (Outline of literature review methodology, including search flow chart and document search database). [file 12889_2021_11257_MOESM1_ESM.docx]

**Additional File 1 – Literature Review Methodology**

### **Methodology of document review**

A review of academic literature, grey literature and Nepal-specific policy documents was undertaken to inform and supplement interviews. Preliminary EMBASE and MEDLINE database searches to optimise search terms and determine scope revealed a dearth of literature specific to Nepal implementing WHO Marketing Recommendations. The review was thus broadened to include literature covering: marketing of unhealthy foods in Nepal and other LMICs [86]; marketing of tobacco, alcohol and BMS in Nepal (acknowledging similar challenges and opportunities, but differences such as how these risk factors are governed globally [87]); and generally opportunities and barriers to addressing NCDs in Nepal. Search terms were applied consistently alongside related subject headings across social sciences, public policy and global health databases (**Table 1**), with grey literature source websites searched using Key Terms in June 2018. Reference list scanning and academics were contacted to identify literature not retrieved.

The search strategy and application of eligibility criteria (**Table 1**), identified 1226 documents for screening (**Figure 1**). This led to in-depth review of 166 documents, with 37 included in the final analysis. A list of studies is included (**Table 2**) for reliability [37].

**Table 1 – Search terms, Data sources, Inclusion criteria**

| **Search terms** | |
| --- | --- |
| **Key terms** | **Search terms** |
| Nepal | Nepal* |
| LMIC | low- and middle-income countr* OR LMIC* OR LICs OR low-income countr* OR MICs* OR middle-income countr* OR developing countr* OR Asia* OR Brazil* OR Mexico* OR India* OR Malaysia* OR South Africa* OR Iran* OR Fiji* OR Philippine* OR Thailand* OR Costa Rica* OR Peru* OR Turkey* |
| Marketing | market* OR advert* |
| Unhealthy foods or beverages | unhealth* OR junk OR HFSS OR high fat salt* sugar* OR high salt OR high sugar OR trans-fat* OR ultra-processed |
| Tobacco, alcohol, BMS | tobacco* OR alcohol* OR breast milk substitute* OR formula milk OR breast-feeding |
| NCDs | NCD* OR non-communicable disease* OR noncommunicable disease* OR nutrition |
| Opportunities and barriers | barrier* OR opportunit* OR influenc* OR facilitat* OR political commit* OR policy OR policies OR regulat* |
| **Data sources** | |
| **Scholarly literature** | ProQuest Central; EMBASE; IBSS; MEDLINE; SCOPUS; Web of Science |
| **Grey literature, including laws and policies** | DFID; Food and Agricultural Organisation of the UN; Institute for Development Studies; International Food Policy Research Institute; McCabe Centre for Law and Cancer; Nepal government websites, including the Nepal Law Commission [88] and Nepal Nutrition and Food Security Portal [89]; Overseas Development Institute; Save the Children; Scaling-up Nutrition WHO, including the Global Database on the Implementation of Nutrition Action (GINA); UNICEF; UN System Standing Committee on Nutrition; USAID; World Bank; World Cancer Research Fund International; World Food Programme |
| **Inclusion criteria** | |
| **Inclusion** | 1. Published after 2000 in English, before the Framework Convention on Tobacco Control (FCTC) was adopted [90] 2. Full article accessible 3. Considers policy change in the area, or implementation, of WHO Marketing Recommendations, NCD policies or restrictions on marketing of tobacco, alcohol or BMS (as relevant), or factors influencing these processes 4. Considers experiences in LMICs (including Upper Middle-Income Countries) that are relevant to the case under analysis 5. Methodology largely acceptable, including published commentaries from reputable sources including global organisations, civil society and research institutes (noting the later are largely grey literature) |
| **Exclusion** | Converse of above, including only focuses on HICs |

**Figure 1** – **Search Flow Chart**


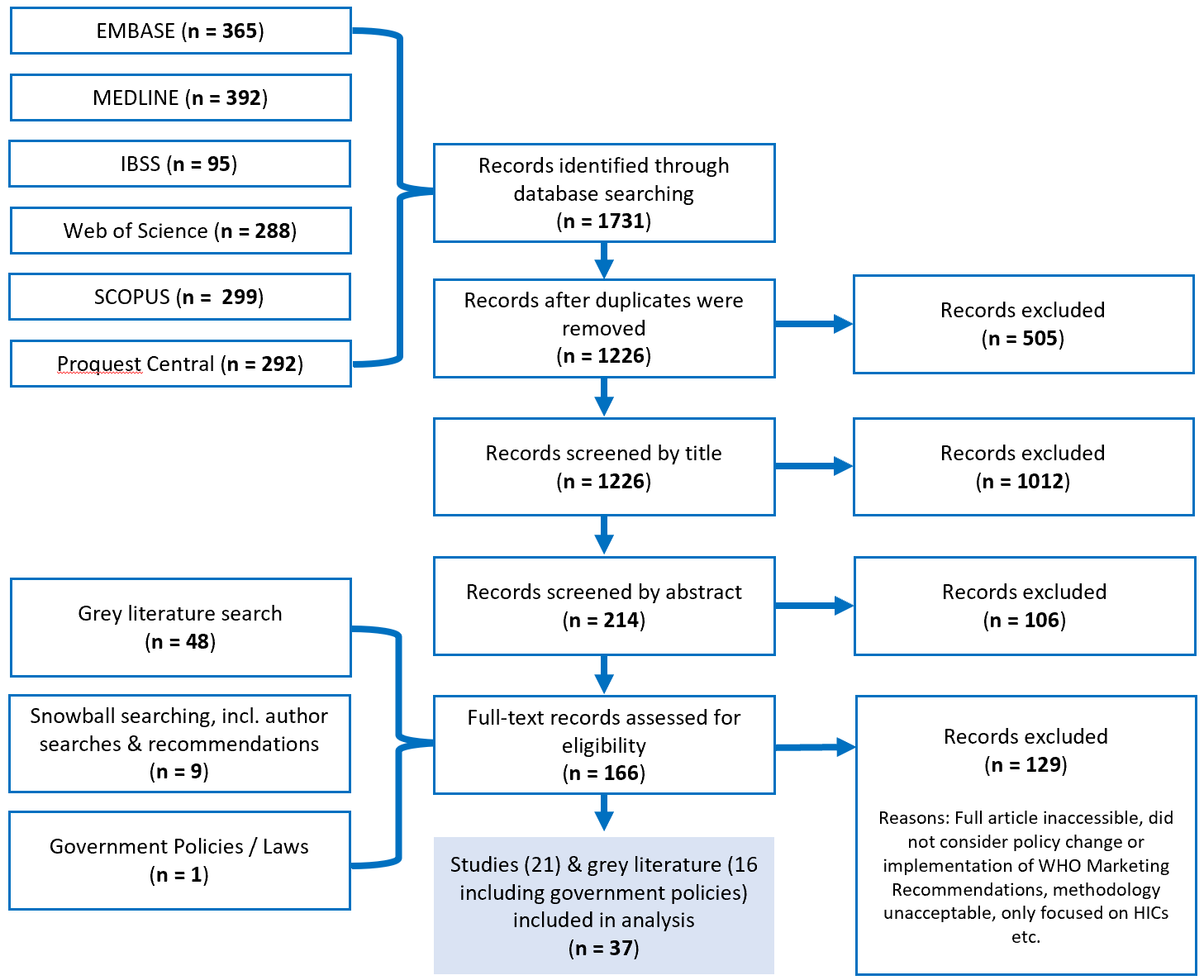


**Table 2 – Document search database**

| **Type** | **No.** | **Document description** |
| --- | --- | --- |
| **Academic papers** | 1 | BARQUERA, S. 2017. What the world can learn from Mexico's battle against obesity: Vulnerable groups, international collaboration, evidence-based policies and management of conflicts of interest. *Annals of Nutrition and Metabolism,* 71**,** 14. |
|  | 2 | BARQUERA, S., CAMPOS, I. & RIVERA, J. A. 2013. Mexico attempts to tackle obesity: The process, results, push backs and future challenges. *Obesity Reviews,* 14**,** 69-78. |
|  | 3 | DELOBELLE, P. M. D. P., SANDERS, D. M. D. C. H. M. D., PUOANE, T. M. P. H. D. & FREUDENBERG, N. D. 2016. Reducing the Role of the Food, Tobacco, and Alcohol Industries in Noncommunicable Disease Risk in South Africa. *Health Education and Behavior,* 43**,** 70. |
|  | 4 | FRASER, B. 2013. Latin American countries crack down on junk food. *The Lancet,* 382**,** 385-6. |
|  | 5 | GOMES, F. S. 2010. Regulation of marketing of food and non-alcoholic beverages in Brazil. *Obesity Reviews,* 11**,** 63. |
|  | 6 | HAWKES, C. & LOBSTEIN, T. 2011. Regulating the commercial promotion of food to children: A survey of actions worldwide. *Int. J. Pediatr. Obes.,* 6**,** 83-94. |
|  | 7 | JAACKS, L. M., KAVLE, J., PERRY, A. & NYAKU, A. 2017. Programming maternal and child overweight and obesity in the context of undernutrition: current evidence and key considerations for low- and middle-income countries. *Public Health Nutr,* 20**,** 1286-1296. |
|  | 8 | JAIME, P. C., DA SILVA, A. C., GENTIL, P. C., CLARO, R. M. & MONTEIRO, C. A. 2013. Brazilian obesity prevention and control initiatives. *Obes Rev,* 14 Suppl 2**,** 88-95. |
|  | 9 | MOISE, N., CIFUENTES, E., OROZCO, E. & WILLETT, W. 2011. Limiting the consumption of sugar sweetened beverages in Mexico's obesogenic environment: A qualitative policy review and stakeholder analysis. *Journal of Public Health Policy,* 32**,** 458-75. |
|  | 10 | NEUPANE, D. & KALLESTRUP, P. 2013. Non-communicable Diseases in Nepal: Challenges and Opportunities. *J Nepal Health Res Counc,* 11**,** 225-8. |
|  | 11 | PHULKERD, S., SACKS, G., VANDEVIJVERE, S., WORSLEY, A. & LAWRENCE, M. 2017. Barriers and potential facilitators to the implementation of government policies on front-of-pack food labeling and restriction of unhealthy food advertising in Thailand. *Food Policy,* 71**,** 101-110. |
|  | 12 | POPKIN, B., MONTEIRO, C. & SWINBURN, B. 2013. Overview: Bellagio Conference on Program and Policy Options for Preventing Obesity in the Low- and Middle-Income Countries. *Obesity Reviews,* 14**,** 1-8. |
|  | 13 | PRIES, A. M., HUFFMAN, S. L., ADHIKARY, I., UPRETI, S. R., DHUNGEL, S., CHAMPENY, M. & ZEHNER, E. 2016. High consumption of commercial food products among children less than 24 months of age and product promotion in Kathmandu Valley, Nepal. *Matern Child Nutr,* 12 Suppl 2**,** 22-37. |
|  | 14 | REEVE, E., THOW, A. M., BELL, C., ENGELHARDT, K., GAMOLO-NALIPONGUIT, E. C., GO, J. J. & SACKS, G. 2018. Implementation lessons for school food policies and marketing restrictions in the Philippines: A qualitative policy analysis. *Globalization and Health,* 14**,** 8. |
|  | 15 | SHARMA, S. R., PAGE, R., MATHESON, A., LAMBRICK, D., FAULKNER, J. & MISHRA, S. R. 2017. Non-communicable disease prevention in Nepal: systemic challenges and future directions. *Glob Health Promot***,** 1757975917720800. |
|  | 16 | SMITH, R. & IRWIN, R. 2016. Measuring success in global health diplomacy: lessons from marketing food to children in India. *Globalization and health,* 12**,** 28. |
|  | 17 | SOMASUNDARAM, N. P. & KALUPAHANA, N. S. 2016. Population-based dietary approaches for the prevention of noncommunicable diseases. *WHO South-East Asia journal of public health,* 5**,** 22-26. |
|  | 18 | STUCKLER, D., MCKEE, M., EBRAHIM, S. & BASU, S. 2012. Manufacturing Epidemics: The Role of Global Producers in Increased Consumption of Unhealthy Commodities Including Processed Foods, Alcohol, and Tobacco. *PLoS Medicine,* 9**,** e1001235. |
|  | 19 | UPRETI, S. R., LOHANI, G. R., MAGTYMOVA, A. & DIXIT, L. P. 2016. Strengthening policy and governance to address the growing burden of diabetes in Nepal. *WHO South-East Asia journal of public health,* 5**,** 40-43. |
|  | 20 | VAIDYA, A., SHAKYA, S. & KRETTEK, A. 2010. Obesity prevalence in Nepal: public health challenges in a low-income nation during an alarming worldwide trend. *Int J Environ Res Public Health,* 7**,** 2726-44. |
|  | 21 | WORSLEY, A., WANG, W. C., SARMUGAM, R., PHAM, Q., FEBRUHARTANTY, J. & RIDLEY, S. 2018. Household food providers’ attitudes to the regulation of food marketing and government promotion of healthy foods in five countries in the Asia Pacific region. *British Food Journal,* 120**,** 1236-1249. |
| **Grey literature** | 1 | FAO/WHO (2018). Proceedings of the FAO/WHO international symposium on sustainable food systems for healthy diets and improved nutrition. Rome, Italy, FAO/WHO. |
|  | 2 | IFPRI (2015). Global Nutrition Report 2015: Actions and Accountability to Advance Nutrition & Sustainable Development. Washington, D.C., USA, International Food Policy Research Institute (IFPRI). |
|  | 3 | RECPHEC 2014. Rapid Assessment on Media Coverage of Junk Food and its Content Analysis on Selected Nepali and Indian Television Channels. Kathmandu, Nepal: Resource Centre for Primary Health Care (RECPHEC). |
|  | 4 | WHO-EMRO (2016). Summary report on the expert meeting to finalize a regional roadmap to address unopposed marketing of unhealthy foods/beverages to children in the Eastern Mediterranean Region, Cairo, Egypt, 21–22 June 2016. Cairo, Egypt, World Health Organization (WHO), Regional Office for the Eastern Mediterranean (EMRO). |
|  | 5 | WHO-EURO (2014). Seminar on working across sectors For noncommunicable diseases (NCD): Policy responses to marketing of Alcohol and food marketing to children. Copenhagen, Denmark, WHO Regional Office for Europe. |
|  | 6 | WHO-GCM/NCD (2016). Final report and recommendations from the Working Group on ways and means of encouraging Member States and non-State actors to realize the commitment included in paragraph 44 of the Political Declaration of the High-level Meeting of the United Nations General Assembly on the Prevention and Control of Noncommunicable Diseases. Geneva, Switzerland, WHO. |
|  | 7 | WHO-Nepal (2018). "Non-Communicable Diseases." Retrieved 14 June, 2018, from <http://www.searo.who.int/nepal/documents/NEP_NCD/en/>. |
|  | 8 | WHO-SEARO (2009). Strengthening Partnerships for Integrated Prevention and Control of Noncommunicable Diseases: The SEANET-NCD Meeting Report of the Meeting, Chandigarh, India, 15–19 June 2009. India, World Health Organization (WHO) Regional Office for South-East Asia (SEARO). |
|  | 9 | WHO-SEARO (2011). Health and development challenges of noncommunicable diseases in the South-East Asia Region: Report of the regional meeting, Jakarta, Indonesia, 1-4 March 2011. India, World Health Organization (WHO) Regional Office for South-East Asia (SEARO). |
|  | 10 | WHO-SEARO (2015). Approaches to establishing country-level, multisectoral coordination mechanisms for the prevention and control of noncommunicable diseases. India, World Health Organization (WHO) Regional Office for South-East Asia (SEARO). |
|  | 11 | WHO-SEARO (2015). Strengthening NCD Civil Society Organizations: Report of the Regional Meeting, New Delhi, India, 9-10 July 2015. India, World Health Organization (WHO) Regional Office for South-East Asia (SEARO). |
|  | 12 | WHO-WPRO (2013). Informal Consultation on Reducing the Harmful Impact on Children of Marketing Foods, Beverages, Tobacco and Alcohol, Manila, Philippines, 25-26 September 2013: meeting report. Manila, World Health Organization (WHO), Regional Office for the Western Pacific (WPRO). |
|  | 13 | WHO-WPRO (2015). Biregional Workshop on Restricting the Marketing of Foods and Non-Alcoholic Beverages to Children in the Western Pacific and South-East Asia, Kuala Lumpur, Malaysia, 1 to 4 December 2015: meeting report. Manila, World Health Organization (WHO), Regional Office for the Western Pacific (WPRO). |
|  | 14 | WHO (2006). Marketing of food and non-alcoholic beverages to children: report of a WHO forum and technical meeting, Oslo, Norway, 2-5 May 2006. Geneva, Switzerland, WHO. |
|  | 15 | WHO (2008). Scaling up prevention and control of noncommunicable diseases: The SEANET-NCD meeting: Report of the Meeting, Phuket, Thailand, 22–26 October 2007. New Delhi, India, WHO Regional Office for South-East Asia. |
| **Government documents & laws** | 1 | GoN & WHO 2014. Multisectoral Action Plan for the Prevention and Control of Non-Communicable Diseases (2014-2020). Nepal: Government of Nepal. |
