# Supplementary material for: Barriers and opportunities to restricting marketing of unhealthy foods and beverages to children in Nepal: a policy analysis
Source: BMC Public Health. 2021 Jul 8;21:1351. doi: 10.1186/s12889-021-11257-y (PMC8268610; doi:10.1186/s12889-021-11257-y)
Supplement: Supplementary file 2 — Additional file 2. Respondent Database (List of study respondents and respondent type) and Policy Document Review. [file 12889_2021_11257_MOESM2_ESM.docx]

## **Additional File 2 – Respondent database & Policy document review**

**Respondent database**

| **Respondents** | 1 |  | GOV Civil service (including health policy-makers and regulators) |
| --- | --- | --- | --- |
|  | 2 | | PS Private sector |
|  | 3 | | CSO Civil society |
|  | 4 | | RES Health research |
|  | 5 | | PS Private sector |
|  | 6 | | GOV Civil service (including health policy-makers and regulators) |
|  | 7 | | IO International organisation |
|  | 8 | | GOV Civil service (including health policy-makers and regulators) |
|  | 9 | | GOV Civil service (including health policy-makers and regulators) |
|  | 10 | | IO International organisation |
|  | 11 | | RES Health research |
|  | 12 | | IO International organisation |
|  | 13 | | DR Clinician |
|  | 14 | | MED Media industry (regulation) |
|  | 15 | | GOV Civil service (including health policy-makers and regulators) |
|  | 16 | | RES Health research |
|  | 17 | | CSO Civil society |
|  | 18 | | MED Media industry (private) |

**Policy document review**

- ***Nepal’s health sector policies and legislation***
  - Mother’s Milk Substitutes (Control of Sale and Distribution) Act, 2049 (1992)
  - Multisectoral Action Plan for the Prevention and Control of NCDs 2014–2020
  - Multi-sector Nutrition Plan For Accelerated Reduction of Maternal and Child Under-nutrition in Nepal 2013-2017
  - National Health Policy 2014
  - National Health Policy 1991
  - National Nutrition Policy and Strategy 2004
  - National Safe Motherhood and Newborn Health Long Term Plan (NSMNH-LTP) 2006-2017
  - National School Health Nutrition Strategy Nepal, 2006
  - Nepal Health Sector Strategy (2015-2020)
  - Second Long Term Health Plan 1997-2017
  - The Mother's Milk Substitutes (Control of Sale and Distribution) Regulation, 1994 (2051)
- ***Nepal’s non-health sector policies***
  - Constitution of Nepal (2015)
  - Consumer Protection Act 2054 (1998)
  - Education Act, 2028 (1971)
  - Food Act 2023 (1967)
  - Food and Nutrition Security Plan of Action (FNSP) - A National Programme for Food and Nutrition Security 2013
  - Food Rules, 2028 (1970)
  - Industrial Policy 2010
  - Media Policy 2012
  - Multi-sector Nutrition Plan II (2018-2022)
  - National Adolescent Health & Development Strategy 2000
  - National Plan of Action for Children: Nepal (2004/05 – 2014/15)
  - The National Broadcasting Act, 1993
  - Trade Policy 2015
