# Supplementary material for: Barriers and opportunities to restricting marketing of unhealthy foods and beverages to children in Nepal: a policy analysis
Source: BMC Public Health. 2021 Jul 8;21:1351. doi: 10.1186/s12889-021-11257-y (PMC8268610; doi:10.1186/s12889-021-11257-y)
Supplement: Supplementary file 3 — Additional file 3. Interview Guide for Stakeholders. [file 12889_2021_11257_MOESM3_ESM.docx]

**Additional File 3 – Interview Guide for Stakeholders**

# Basis of Interview Guideline for Stakeholders

# CREHPA/UCL, 2018

**Interviews are based on Baker et al’s framework for driving political commitment for nutrition. Paper available here:** Baker P, Hawkes C, Wingrove K*, et al*

What drives political commitment for nutrition? A review and framework synthesis to inform the United Nations Decade of Action on Nutrition

*BMJ Global Health*2018;**3:**e000485

| **Baker et al.’s Framework for driving political commitment for nutrition** | |
| --- | --- |
| **Category** | **Factor and description** |
| **Actors** | **(1) Nutrition actor network (NAN) effectiveness:** effectiveness of NANs, the individuals and organisations operating within a given jurisdiction who shared common principles, causal beliefs and/or interest in tackling malnutrition and who acted collectively to do so. |
|  | **(2) Strength of leadership:** presence of committed and politically savvy individuals, within or outside of government, recognised as strong champions for nutrition. |
|  | **(3) Civil society mobilisation**: extent to which civil society groups mobilised to address malnutrition, including non-government organisations and social movements collectively representing the interests of citizens. |
|  | **(4) Supportive international actors:** degree to which actors with an international scope of operations and/or membership initiated, championed and/or supported nutrition policy and programming responses. |
|  | **(5) Private sector interference:** degree to which mobilised private interest groups undermined effective nutrition policy responses, including food producers, retailers, marketers and their representative peak bodies. |
| **Institutions** | **(6) Strength of institutions:** extent to which coordinating agencies and institutional systems mandated to address malnutrition were empowered to effectively coordinate multisector/multilevel responses and advocate for sustained attention and resources. |
|  | **(7) Effective vertical coordination:** degree to which nutrition policies were effectively coordinated, implemented and monitored across levels of governance, particularly regarding the incentives of subnational actors to adopt, progress and benefit from central government policies. |
|  | **(8) Legislative, regulatory and policy frameworks**: degree to which national nutrition policies, operational plans and enabling legislation were well-designed and enacted, and/or the alignment of nutrition objectives with broader policy agendas and regulatory frameworks. |
| **Political and societal contexts** | **(9) Supportive political administrations:** degree to which members of the executive (e.g., head of state, ministers), legislative (e.g., parliamentarians) and administrative (e.g., agency heads, senior officials) branches of government initiated and championed nutrition responses. |
|  | **(10) Societal conditions and focusing events:** extent to which changing societal conditions (long-duration phenomena) or focusing events (short-term processes) focused attention onto nutrition or closely related issues and presented opportunities or impediments to commitment-building. |
|  | **(11) Ideology and institutional norms:** extent to which entrenched belief systems and practices predominant within political systems, policy-making institutions and/or in society-at-large, negatively skewed perceptions about malnutrition problems and undermined effective policy responses. |
| **Knowledge, evidence and framing** | **(12) Credible indicators and data systems:** availability of credible indicators and high-quality data systems for monitoring nutrition problems, informing policy design, tracking progress and empowering accountability systems. |
|  | **(13) Evidence:** extent to which robust evidence on the causes, manifestations and consequences of malnutrition and the efficacy and cost-effectiveness of interventions was available, clearly communicated and accepted. |
|  | **(14) Internal frame alignment:** degree to which NANs were aligned around a common interpretation and narrative of a given malnutrition problem including its definition, magnitude, causes and solutions for resolving it. |
|  | **(15) External frame resonance:** degree to which NANs publicly portrayed (i.e. framed) nutrition problems and solutions in ways that resonated with and motivated action by external audiences, and countered the frames deployed by opponents. |
| **Capacities and resources** | **(16) Strategic capacities:** degree to which NAN members possessed ‘soft-power’ skills including the capacity to generate consensus, resolve conflicts, respond to recurring opportunities and challenges, build strategic alliances, undertake strategic communications and related tasks. |
|  | **(17) Organisational capacities:** degree to which NAN members possessed the technical knowledge and skills, administrative systems and human resources required to generate commitment, including through the effective management of nutrition policy and programming responses. |
|  | **(18) Financial resources:** degree to which nutrition budgetary commitments and financing systems incentivised multisector/multilevel coordination, ensured successful policy implementation and created ownership and entitlements among political elites, policy-makers, citizens and other stakeholders. |

**Generic guide**

**Problem characteristics -** covering Knowledge, evidence & framing

- Do you think that sugar/salt/transfats is a particularly important problem for Nepal?
- What are your views on the relationship between these products and health?

**Policy content -** covering Knowledge, evidence & framing

- What do you think that the policy (on sugar, salt or transfat) 1) consists of; and 2) aims to achieve.
- What is the underlying problem that the policy is addressing? – Probe, is it individual behavior or industry profit?
- In your view is this the appropriate policy response?
- Why do you think human rights approaches were not included in the policy?
- Why or why not were vulnerable groups targeted in the policy?

**Who or what drove the policy on** – covering Political and societal contexts, Institutions and Actors

- Who put the issue on the agenda, or proposed those particular contents?
- Was there a particular event that triggered it?
- Was there opposition to putting it on the agenda or formulating a policy? What was the source of the opposition?
- What specific interests were these actors (pro or against) pursuing or protecting?
- As a result of these interests, how committed/passionate were the actors in relation to their position on the policy?

**Actors –** covering Actors, Institutions and Capacities and resources

1. **Individuals/organisations**. Who were key actors in the policy process, what are their major sources of influence on the process (and policy content)

– probe - is it because of their formal positions (administrative/regulatory/political/legal), charisma, rhetorical skills (discursive and ability to frame the problem), resources (financial, network base), access to media, other?

1. **Nutrition Actor Networks**. Thinking about the key actors identified above, are some of them connected through formal and informal networks? Did these networks enable them to have more or less common positions?
2. **Guiding institutions**. Can you identify a key organization or coordination platform that took leadership on the issue?
3. **Leadership**. Were there key champions (individuals or organisations) for the issue and/or policy?
4. **Civil society**. Is it mobilized around the issue or policy?
5. **International actors**. Were there any that played a particular role?

**Framing** – covering Knowledge, evidence and framing and Capacities and resources

- What were the arguments used by the key actors about the importance of the problem and how to solve it?
- Is there a more or less common perception of the problem and or policy solution (e.g., for or against the policy)?
- What arguments and ideas were put forward in terms of policy beneficiaries or losers (i.e., costs and benefits of the policy to particular communities and populations)?

**Process and Budget allocation and implementation –** covering Capacities and resources and Institutions

- What was the process through which the policy emerged?
- What was your role in the policy process?
- Who was the key driver?
- Can you tell me about the budget allocated for the policy and/or the multi sectoral action plan on prevention and control of NCD?
- What are your thoughts on implementation of the policy – and their effectiveness?
